# Stigmatizing attitudes towards people living with HIV/AIDS: validation of a measurement scale

**DOI:** 10.1186/1471-2458-14-1246

**Published:** 2014-12-04

**Authors:** Marianne Beaulieu, Alix Adrien, Louise Potvin, Clément Dassa, Comité consultatif sur les attitudes envers les PVVIH

**Affiliations:** Département de médecine sociale et préventive, Université de Montréal, Montréal, Canada; Secteur Vigie et Protection, Agence de la santé et des services sociaux de Montréal, Montréal, Canada

**Keywords:** Attitudes, Stigma, HIV/AIDS, Scale, Validation

## Abstract

**Background:**

Although stigmatization has long been recognized as a major obstacle to HIV prevention. The lack of a valid and reliable measurement tool for stigmatization is a major gap in the research. This study aimed to: 1) develop a scale of stigmatizing attitudes towards people living with HIV (SAT-PLWHA-S) and 2) demonstrate its reliability and validity.

**Methods:**

French and English-speaking experts (n = 21) from different professional communities (academics, practitioners) assessed the clarity and relevance of the proposed items. The psychometric properties of the SAT-PLWHA-S were assessed with a random digit dial population based telephone survey (n = 1,500) of respondents in Quebec, Canada. Analyses included exploratory and confirmatory factor analyses, correlations, multiple linear regressions, t-tests, hypothesis testing of factorial structure invariance, and Cronbach’s alpha.

**Results:**

Confirmatory factor analysis (CFA) supported a 27-item structure with seven factors: 1) concerns about occasional encounters; 2) avoidance of personal contact; 3) responsibility and blame, 4) liberalism, 5) non-discrimination, 6) confidentiality of seropositive status, and 7) criminalization of HIV transmission. Cronbach’s alphas indicate satisfactory internal consistency. An assessment of concurrent validity using Pearson’s correlation and multiple linear regression shows that homophobia and HIV transmission knowledge are significant determinants of stigmatizing attitudes toward PLHIV. Discriminant validity (t-test) results suggest that the SAT-PLWHA-S can differentiate attitudes between different groups and indicates invariant factor structure across language.

**Conclusions:**

The results of this study suggest that the SAT-PLWHA-S is a reliable and valid tool for measuring stigmatizing attitudes toward PLHIV and that it can contribute to a deeper understanding of HIV stigma.

**Electronic supplementary material:**

The online version of this article (doi:10.1186/1471-2458-14-1246) contains supplementary material, which is available to authorized users.

## Background

Since the first cases were identified in the early 1980s HIV/AIDS has become epidemic. In Canada, there were an estimated 71,300 people living in 2011 with HIV/AIDS (PLWHA), one-quarter of whom did not know of their serological status [1]. HIV/AIDS stigmatization poses a major challenge to preventive public health efforts by contributing to underreporting of cases [2–5]. Early detection is therefore key to preventing the spread of HIV [6] because it encourages individuals to adopt safer practices [7–16] and results in more effective and efficient medical care by reducing the infectivity of individuals with HIV [17–21], and therefore the risk of spreading the virus [22–24]. Even when they are aware of the risks of infection, many people avoid taking a screening test for fear that the result will be positive and they will have to cope with the stigma that accompanies the disease [2, 3, 25–31]. Therefore, among the harmful consequences of this stigma is that it contributes to the spread of the disease [26, 32].

Stigma is defined as “an attribute that is deeply discrediting” [33]. Stigmatization is also defined as an intertwined mix of perspective (perceiver vs. target), identity (group-based vs. personal) and cognitive-affective-behavioral response [34]. The current study is interested in the cognitive-affective-behavioral responses of perceivers. Sociocognitive conceptualization of stigmatization operationalize these responses with three theoretical components: stereotyping (cognitions), prejudice (affects) and discrimination (behaviors) [35–39].

There are several scales in the literature measuring different aspects of the stigmatization of PLWHA in various areas of the world [40–48]. The cognitive dimension (e.g. stereotypes) is the most frequently studied dimension [40, 42–45, 47, 48]. These generally capture moral judgment (e.g. PLWHA should be blamed, punished, condemned or held responsible for being HIV-positive) or the expression of negative beliefs towards groups disproportionately affected by the epidemic (e.g. being dirty or cursed) [3, 44, 49–52]. The second most documented dimension is the behavioral one (e.g. discrimination) [40, 42, 44, 46–48]. It is operationalized as treatment from society, such as whether PLWHA should be supported or discriminated against and whether their behaviour should be circumscribed. There are two other dimensions, interpersonal distancing (e.g. minimizing contact with PLWHA) and use of coercive measures (e.g. confidentiality of serological status, position on HIV/AIDS criminalization) [53]. The affective dimension is rarely measured [40–42, 44, 47, 48]. It relates to feeling favourably towards infected persons or respondents’ comfort in interacting with PLWHA in different settings. Many authors have stressed that HIV/AIDS prejudice can largely be explained by the fears, sometimes irrational, that surround the disease: fear of catching it, its incurable nature, and its potentially lethal consequences [5, 44, 49–51, 54].

One obstacle to understanding the impact of HIV/AIDS stigmatization on the spread of the disease in North America is the scarcity of empirical research in this area [55]. In addition, studies that have attempted to describe and measure HIV/AIDS stigmatization contain certain methodological shortcomings, are generally atheoretical, and fail to cover all its conceptual dimensions [35, 56–58]. There are few published questionnaires in the literature that measure different dimensions of PLWHA stigmatization [40–47, 59]. Dimensions most frequently documented are PLWHA blame, legislative procedures or monitoring of PLWHA and PLWHA rights. Other dimensions of stigma are rarely measured: comfort level with PLWHA, prejudices against PLWHA, feelings towards PLHIV and social support. Most of the HIV stigma measures address prejudices and discrimination, but few have documented the stereotypes. Moreover, measures need to be adapted to account for new realities concerning the disease, particularly since the introduction of antiretroviral treatments (ARV). Consequently, some measures have become outdated and no longer reflect current concerns. For example, in recent years, to measure support for coercive measures, statements addressing the quarantine of PLWHA [60, 61] rather than mandatory disclosure of seropositive status [62] or the criminalization of PLWHA have become more widely used [63].

Although existing questionnaires have all been published, a small number of measures have been examined for validity and reliability [35]. Few studies have used expert consultation [64] to determine the relationship between stigmatization measures and the concepts considered to ensure content validity [53]. Researchers have rarely used advanced methodologies such as confirmatory factor analysis to provide a deeper understanding of the constructs derived from measuring instruments. Among the questionnaires published in the literature, only three have been the subject of rigorous validation analysis [40, 45, 59]. The scale of Kalichman and colleagues’ scale [45] was developed to measure PLWHA stigmatization in South Africa, while the Bresnahan & Zhuang’s scale [59] does not cover all the stigma dimensions. Adrien and colleagues [40] developed their instrument in 1996 and used it in Canada (more specifically, in the province of Quebec). For that reason, Adrien et al. [40] instrument was chosen to serve as a basis for the development of a new measurement scale.

### Context

In spring 2009, the Ministry of Health and Human Services funded the Public Health Department of Montreal to conduct a survey to document the evolution of attitudes towards PLWHA in Quebec. This survey was the third to be conducted in Quebec [40]. The first, conducted in 1996, sought to assess attitudes and risk behaviors associated with HIV in the general population, with a 16 items scale in a sample of 3501 people (5 factors: fear of being infected, fear of contact with PLWHA, prejudicial beliefs toward groups at high risk of HIV, tolerance regarding sexual mores and behaviors, social support for PLWHA). In spring 2002, the same study was repeated to measure changes that may occurred since 1996 in attitudes towards PLWHA. The second study included the scale validated in 1996 (16 items – 5 factors), while adding new statements that take into account the emergence of new attitudes within the population of Quebec on the evolution of the epidemic or new measures to control epidemic.

The aim of this study is to validate the modified version of a scale designed to measure stigmatizing attitudes toward PLWHA (SSAT- PLWHA) adapted to the new reality of HIV/AIDS in the general population of Quebec, Canada.

## Methods

### Development of the SAT-PLWHA-S

The SAT-PLWHA-S was developed in four phases: planning, construction, testing, and validation [65]. The study protocol was approved by the Review Boards of the Faculty of Medicine of Université de Montréal and of the Montreal Public Health governmental authority.

In the planning phase, a steering committee was formed of various stakeholders from clinical, community, decision-making, and university circles concerned with the issue of HIV/AIDS stigma in Quebec. Some of the committee members were living with HIV. Its mandate was to deepen the understanding of current dimensions of HIV/AIDS stigma. Structured literature searches were conducted on various search engines (e.g., PubMed, Sociofile, Google Scholar) which enabled defining dimensions of stigma both conceptually sound and relevant to practice: responsibility and blame, confidentiality of serological status, HIV criminalization, PLWHA coercion, PLWHA rights, negative feelings towards PLWHA [35–38, 41–48, 53]. The following dimensions were added to the existing ones: fear of being infected, fear of contact with PLWHA, prejudicial beliefs toward groups at high risk of HIV, tolerance regarding sexual mores and behaviors, and social support for PLWHA [40].

In the construction phase a pool of items was developed for each dimension of stigma identified in the planning phase, items were formulated by the lead author. A group of Canadian experts (n = 21) was selected according to language spoken (French (n = 13); English (n = 8)) and type of expertise (academics specialized in HIV (n = 9); front line workers, health professionals or public health decision makers (n = 12)). Some of the experts consulted were openly living with HIV. The experts’ mandate was to assess the clarity and relevance of the proposed items to ensure the scale’s content validity. Each item was assessed in relation to a conceptual definition on a seven-point Likert scale from 1 (not relevant/not clear) to 7 (relevant/clear). The experts were also asked to comment on the items and suggest missing questions or dimensions. The responses and comments were reviewed and considered by the research team. No cut-off point was predetermined; the items were compared among themselves on the basis of expert assessment. Items that earned higher scores for relevance and clarity from all expert subgroups (language and type of expertise) were retained in the first version of the questionnaire.

To ensure item clarity, the questionnaire was tested in a convenience sample of 24 respondents using the same method as for the data collection - telephone interviews. Inclusion criteria were age from 15 to 65 years and the ability to speak French. After completing the questionnaire, respondents were asked to comment on the experience and, if appropriate, to explain during a brief semi-structured interview any difficulties they had.

The questionnaire was then administered by a survey firm to 200 respondents (100 French-speaking and 100 English-speaking) as a pretest. The aim was to assess the average duration of the interviews, comprehension of the questions, the logical structure of the questionnaire, and how to program skipped questions and sections. Based on the pretest the questionnaire was again slightly modified.

The polling firm Léger Marketing collected data for the final survey from March 15 to April 2, 2010. Interviews were conducted by experienced interviewers, based on instructions provided by the research team. For the telephone interviews a stratified sample was used, consisting of 1,500 individuals living in all regions of Quebec aged from 15 to 64 years and able to speak French or English. Non-proportional quota sampling was therefore used to build the samples of French and English-speaking participants (French-speaking N = 1,040; English-speaking N = 460). Participants’ telephone numbers were randomly selected using ASDE Survey Sampler. All participants provided oral consent. Respondents were randomly selected within households according to whose birthday came next. The targeted response rate was 73.0% and the obtained response rate was 73.5%.

### Measures

#### Scale of stigmatizing attitudes towards people living with HIV

The final version of the questionnaire on stigmatizing attitudes towards PLWHA contained 42 items (Table 1) covering eight dimensions based on the literature review: concerns about occasional encounters (5 items), avoidance of personal contact (3 items), responsibility and blame (6 items), liberalism (4 items), non-discrimination (7 items), confidentiality of serological status (6 items), position on HIV/AIDS criminalization (6 items), and negative feelings (5 items). The Likert response scale for each statement ranged from 1 (strongly disagree) to 4 (strongly agree). Once validated, some items will be reverse coded such that a higher mean score indicates a more positive overall attitude (continuous score ranging from 1 to 4).

**Table 1:** **Complete questionnaire (42 items)**

	Item labelling
Prejudices (affects)	**1.Being around someone who has AIDS does not bother me.**
	**2.I would not be worried for my health if a co-worker had AIDS.**
	**3.It would not bother me if there was a boarding house for people with AIDS on my street.**
	21.I would quit my job before I would work with a person who is infected by the AIDS virus.
	**23.If I had a roommate and discovered he was infected with the AIDS virus, it would not bother me.**
Prejudices (affects)	**4.** ^**-**^ **I could not be friends with someone who has AIDS.**
	**5.** ^**-**^ **I would limit my contact with a person whom I know is infected with AIDS.**
	**6.** ^**-**^ **I would not hug someone with AIDS.**
Stereotyping (cognitions)	**7.** ^**-**^ **People who use injectable drugs deserve to have AIDS.**
	**8.** ^**-**^ **My support for a person living with AIDS depends on how the person was infected.**
	**9.** ^**-**^ **I am disgusted by persons who were infected during homosexual relations.**
	**17.** ^**-**^ **People who are infected with the AIDS virus because they have not used a condom deserve what they get.**
	**29.** ^**-**^ **People with AIDS have only themselves to blame.**
	**39.** ^**-**^ **Most people with AIDS are responsible for having their illness.**
Values	**10.** ^**-**^ **To fight AIDS, it is necessary that young people not have sex.**
	**11.** ^**-**^ **Reinforcement of traditional sexual values will help to control AIDS.**
	**12.** ^**-**^ **The arrival of AIDS is linked to the fact that people have more sexual freedom.**
	**13.** ^**-**^ **The spread of AIDS is linked to the decline of moral values.**
Discrimination (actions)	**14.People who have AIDS should have the right to work serving the public, as waiters-waitresses, cooks, hairdressers.**
	**15.Children who are infected with the aids virus should be able to go to day-care.**
	**16.Doctors with AIDS should be allowed to go on working with their patients.**
	**19.People infected with the aids virus should be allowed to immigrate to Canada.**
	24.Women who know they are infected with the AIDS virus have the right to have children.
	28.People who are infected with the AIDS virus have the right to have a love life.
	35.People with AIDS have the right to be sexually active.
Discrimination (actions)	18.A doctor should have the right to warn the sexual partners of a person who has the AIDS virus if that person refuses to do so.
	22.If my partner has the AIDS virus, I should be warned, even without his or her permission.
	**27.** ^**-**^ **I have the right to know if someone around me is infected with the AIDS virus.**
	**32.When a screening test indicates that someone is infected with the AIDS virus, the result should remain confidential.**
	38.People infected with the AIDS virus should inform their sexual partners.
	**41.** ^**-**^ **Doctors should report the names of people with AIDS to the government.**
Discrimination (actions)	20.It is criminal for a person who knows that he or she is infected with the AIDS virus not to use a condom.
	**26.** ^**-**^ **Transmitting the AIDS virus should be punishable by law.**
	**31.** ^**-**^ **People who know they are infected with the AIDS virus and who transmit the virus are criminals.**
	**34.** ^**-**^ **Transmitting the AIDS virus is a crime.**
	37.People who know they are infected with the AIDS virus and share their needles with other people should be punished under the law.
	40.Transmitting the AIDS virus is a crime only if done so intentionally.
Prejudices (affects)	25.I am disgusted by people who have the AIDS virus.
	30.I feel compassion for people infected with the AIDS virus.
	33.People who are infected with the AIDS virus are disgusting.
	36.I feel afraid of people with AIDS.
	42.I feel sympathetic towards people who are infected with the AIDS virus.

*F1: Concerns about occasional encounters = items 1-2-3; F2: Avoidance of personal contact = items 4-5-6; F3: Responsibility and blame = items 7-8-9-17-29-39; F4: Liberalism = items 10-11-12-13; F5: Non-discrimination = items 14-15-16-19-23; F6: Confidentiality of serological status = items 27-32-41; F7: Criminalization of transmission = items 26-31-34;* ***Bold*** *items constitute the SAT-PLWHA-S; .* ^*-*^ *reverse-coded items.*

#### Measure of homophobia

The short version of the Attitudes Toward Gay Men scale [66] contained five items. The Likert response scale ranged from 1 (strongly disagree) to 5 (strongly agree). The short scale showed strong correlations with the original scale (*r* = 0.96) and satisfactory reliability (α = 0.87) in a validation study using a telephone survey [66]. Scores on the homophobia scale were obtained by averaging the five item scores (ex. “I think male homosexuals are disgusting”, “Homosexual behavior between two men is just plain wrong”), higher score indicates more homophobia.

#### Measure of HIV/AIDS transmission knowledge

A seven-item scale addressing transmission knowledge was also administered to respondents [40]. For each item respondents were asked to assess the risk of HIV/AIDS transmission on a four-point scale from 1 (very low risk of infection) to 4 (very high risk of infection). Results on HIV/AIDS transmission knowledge were weighted (to reflect a priori experts’ criteria) by coding very high knowledge as 4, relatively high and relatively low partial knowledge as 3 and 2, respectively, and absence of knowledge as 1. The score on this scale was obtained by averaging the scores on the seven items (ex. risk of being infected when… “Sharing a glass with a person infected with the AIDS virus”, “Shaking hands with a person infected with the AIDS virus”), higher score indicates higher knowledge.

#### Sociodemographic measures

The sociodemographic variables considered included sex, language spoken at home (French or English), age, country of birth (Canada or other), number of years of education, and being acquainted with a PLWHA.

### Analysis

In order to validate the SAT-PLWHA-S in the general Quebec population, the data were used for construct, discriminant, and criterion-related validity as well as reliability studies. Construct validity was examined in two steps using factor analyses of the survey data. First, the sample was randomly divided into two subsamples. Principal component analyses (PCA) and exploratory factor analyses (EFA) using principal axis factoring (PAF) with OBLIMIN (oblique) rotation were performed on the first subsample to identify the most valid factors among the 42 scale items. EFA were conducted by successively introducing items by conceptual blocks based on theoretical groupings. Groupings with low factor loadings (<0.30) were excluded one by one. Confirmatory factor analyses were then conducted on the second subsample [67].

Confirmatory factor analyses (CFA) were run by applying the weighted least squares (WLS) estimator to the polychoric correlation matrix, an appropriate method for ordinal data [68]. The Chi-squared test is initially used to estimate model fit. Because this test is sensitive to sample size [69], fit is also assessed with other indices. The Comparative Fit Index (CFI) and the Non-Normed Fit Index (NNFI) compare model fit to that of an independent (nul) model, with a value greater than 0.95 indicating good fit [70]. The Goodness-of-Fit Index (GFI) measures the relative amount of variance and covariance predicted by the model, with a value greater than 0.95 indicating good fit [71]. The Root Mean Square Error of Approximation (RMSEA) is a measure of approximate fit in the population, with a value less than 0.06 indicating good fit [70]. The Standardized Root Mean Square Residual (SRMR) is based on the fitted residuals, and a value of less than 0.09 indicates good fit [70]. Analyses were conducted using LISREL (8.80). Once a satisfactory model presenting a factorial complexity of one was obtained, factor scores were computed for each factor by averaging their items. A total score was also computed by averaging all items retained in the scale.

To ensure that the SAT-PLWHA-S can differentiate groups of respondents, discriminant validity was assessed (using t tests with SAT-PLWHA-S mean score) by comparing upper and lower quartile groups for the continuous variables (age, education, knowledge score, homophobia score) and for language and sex.

Specific structure invariance hypotheses were tested for both linguistic groups, Anglophones (a) and Francophones (f) [68]. Four hypotheses were successively tested with additional constraints added at each step. The first hypothesis (B) tested the invariance of the factor pattern, which includes the number of factors, for the two compared groups (here, factor loadings values could differ). The second hypothesis (C) tested the invariance of factor loadings (Λ_x_^(f)^ = Λ_x_^(a)^) assuming (B). The third hypothesis (D) tested the invariance of the covariance matrix for errors of measurement (Θ_δ_^(f)^ = Θ_δ_^(a)^) assuming (C). The fourth hypothesis (E) tested the simultaneous invariance of factor patterns, loadings, covariance, and variance among errors of measurement and among factors (Φ^(f)^ = Φ^(a)^). The hypothesis testing was conducted using robust maximum-likelihood (RML) estimation because underlying approximate normality did not hold for some polychoric correlations (4% of RMSEA tests of close fit implied a rejection of underlying approximate bivariate normality at 5% level of significance). The analyses produced Chi-square goodness of fit test which were complemented by fit indices CFI, NNFI, GFI, RMSEA, and SRMR, including PNFI, a parsimony normed fit index that adjusts downward for more complex models (with fewer degrees of freedom). These models with increasing constraints were successively tested for improvement of the fit. Analyses were performed with LISREL (8.80).

Criterion-related validity was assessed with Pearson’s correlations between factor and total scores on stigmatizing attitudes towards PLWHA and for homophobia scores for the total sample and the French and English subsamples. Sequential multiple regression on the stigmatizing attitudes towards PLWHA total scores was performed by introducing independent variables into the regression model in three successive blocks: 1) homophobia, 2) HIV/AIDS transmission knowledge, and 3) sociodemographic characteristics.

To assess the internal consistency of the total and factor scores identified in the factor analyses, Cronbach’s alphas were calculated using SPSS (17.0). Values greater than 0.70 indicate satisfactory internal consistency [72].

## Results

### Participants’ characteristics

The analyses presented in this study were performed on the responses of 1,370 participants. Respondents for whom data were missing on the SAT-PLWHA-S were excluded from the analyses (n = 130). Once weighted, the total sample size was 1,387 participants. The post-stratification weight scheme adjusted for sex, age, region, and language. Average age of respondents was 41.5 years (SD 13.7 years), and 49.7% were women. Most respondents were relatively educated, with an average of 14.2 years of education (SD 3.4 years). 80.3% of the sample spoke French, reflecting the oversampling among Anglophones, and 89.2% were born in Canada. A quarter of the sample (26.0%) knew a PLWHA.

### Exploratory factor analysis

The EFA performed on the SAT-PLWHA-S scores produced a 27-item (from an initial pool of 42 items) solution grouped into seven factors (see Additional file 1). Concerns about occasional encounters (F1, 3i) measures the discomfort related to occasional interaction. Avoidance of personal contact (F2, 3i) refers to discomfort of physical proximity to a PLWHA. Responsibility and blame (F3, 6i) are stereotypical negative beliefs about PLWHA, in general, and also about the behaviors of groups that are at greater risk of acquiring HIV/AIDS. Liberalism (F4, 4i) captures perceived associations between HIV/AIDS and sexual values, operationalized through morality and sexual norms. Non-discrimination (F5, 5i) reflects the desire to integrate PLWHA into diverse areas of social and professional life, whereas confidentiality of serological status (F6, 3i) and criminalization of HIV transmission (F7, 3i) measure support for coercive measures. Fifteen items with low communalities or factor loadings (<0.30) were excluded. One communality was high (0.70), 24 were moderate (from 0.68 to 0.31), and two were marginally low (0.28). The majority of factor loadings were high (from 0.71 to 0.82) or moderate (from 0.65 to 0.34).

The seven factors had a factorial complexity of 1, indicating a simple structure. The high Kaiser-Meyer-Olkin index (KMO = 0.91) and the significant Bartlett’s test of sphericity (p < 0.001) indicated that the correlation matrix was adequate for EFA. The seven-factor PAF solution explained 43.5% of the total variance and accounted for 75.5% of the variance when the data were reduced to seven dimensions using PCA. Estimated correlations between the factors were moderate (from 0.56 to 0.33) to low (from 0.29 to 0.17).

### Confirmatory factor analysis

The model with seven correlated factors, determined through EFA on the first subsample, was confirmed in the second subsample (n = 689). Results of the CFA on the second subsample are presented in Tables 2 and 3. For the WLS estimation the χ^2^ test of fit was significant, (χ^2^ = 1083.59, df = 303, p < 0.001). According to established procedure, model fit was then assessed with various fit indices [73], revealing good fit (CFI = 0.953; NNFI = 0.945; GFI = 0.969; RMSEA = 0.0612; SRMR = 0.164). As an alternative to WLS, all analyses were rerun using robust RML estimation to correct for lack of normality. These results also indicated good model fit, with SRMR below the 0.09 threshold (see Table 4).

**Table 2:** **Confirmatory factor analysis of the SAT-PLWHA-S: final solution, completely standardized for 7 oblique factors**

Item no.		Regression coefficients*							Error variance
	Item description	F1	F2	F3	F4	F5	F6	F7	
	**F1**: Concerns about occasional encounters								
1	Being around someone who has AIDS does not bother me.	.983							.034
2	I would not be worried for my health if a co-worker had AIDS.	.935							.126
3	It would not bother me if there was a boarding house for people with AIDS on my street.	.796							.366
	**F2**: Avoidance of personal contact								
4	I could not be friends with someone who has AIDS.		.908						.176
5	I would limit my contact with a person whom I know is infected with AIDS.		.888						.212
6	I would not hug someone with AIDS.		.968						.064
	**F3**: Responsibility and blame								
7	People who use injectable drugs deserve to have AIDS.			.802					.357
8	My support for a person living with AIDS depends on how the person was infected.			.822					.325
9	I am disgusted by persons who were infected during homosexual relations.			.956					.086
17	People who are infected with the AIDS virus because they have not used a condom deserve what they get.			.872					.240
29	People with AIDS have only themselves to blame.			.799					.362
39	Most people with AIDS are responsible for having their illness.			.721					.481
	**F4**: Liberalism								
10	To fight AIDS, it is necessary that young people not have sex.				.783				.387
11	Reinforcement of traditional sexual values will help to control AIDS.				.762				.419
12	The arrival of AIDS is linked to the fact that people have more sexual freedom.				.789				.378
13	The spread of AIDS is linked to the decline of moral values.				.787				.380
	**F5**: Non-discrimination								
14	People who have AIDS should have the right to work serving the public, as waiters-waitresses, cooks, hairdressers, etc.					.901			.189
15	Children who are infected with the aids virus should be able to go to day-care.					.857			.266
16	Doctors with AIDS should be allowed to go on working with their patients.					.674			.546
19	People infected with the aids virus should be allowed to immigrate to Canada.					.724			.475
23	If I had a roommate and discovered he was infected with the AIDS virus, it would not bother me.					.821			.326
	**F6**: Confidentiality of serological status								
27	I have the right to know if someone around me is infected with the AIDS virus.						.766		.413
32	When a screening test indicates that someone is infected with the AIDS virus, the result should remain confidential.						.638		.592
41	Doctors should report the names of people with AIDS to the government.						.665		.558
	**F7**: Criminalization of transmission								
26	Transmitting the AIDS virus should be punishable by law.							.686	.529
31	People who know they are infected with the AIDS virus and who transmit the virus are criminals.							.692	.521
34	Transmitting the AIDS virus is a crime.							.960	.079

*Note.* n = 689. *All regression coefficients and error variances are significant at 0.05.

**Table 3:** **Confirmatory factor analysis of the SAT-PLWHA-S: completely standardized correlations among factors**

Factors	F1	F2	F3	F4	F5	F6	F7
F1: Concerns about occasional encounters	1.00						
F2: Avoidance of personal contact	.850	1.00					
F3: Responsibility and blame	.712	.767	1.00				
F4: Liberalism	.560	.612	.790	1.00			
F5: Non-discrimination	.753	.868	.755	.652	1.00		
F6: Confidentiality of serological status	.567	.524	.616	.648	.651	1.00	
F7: Criminalization of transmission	.268	.289	.386	.512	.636	.507	1.00

*Note.* n = 689.

**Table 4:** **Confirmatory factor analysis of the SAT-PLWHA-S: fit indices of the confirmatory factor analysis according to the estimator used**

Estimator	χ2	df	p	CFI	NNFI	GFI	RMSEA	SRMR
WLS	1083.59*	303	0.001	0.953	0.945	0.969	0.0612	0.164
RML	504.41*	303	0.001	0.992	0.991	0.882	0.0311	0.0504

*Note.* n = 689. df = degrees of freedom; CFI = comparative fit index; NNFI = non-normed fit index; GFI = goodness-of-fit index; RMSEA = root-mean-square error of approximation; SRMR = standardized root-mean-square residual. *p < 0.05.

### Factor structure invariance

Table 5 presents the four hypothesis tests (B, C, D, and E) for equality of factor structures in French and English speakers. The analysis of fit indices and chi-square change (and its level of significance) indicate good fit and confirm that the scale has the same factor pattern (Hypothesis B), factor loadings (Hypothesis C), variances and covariances of errors of measurement (Hypothesis D), and factor variances and covariances (Hypothesis E) for French and English speakers. As changes in chi-square results are sensitive to sample size, when the sample is large, it is necessary to take into account other criteria such as the change in RMSEA or the change in CFI [74]. As indicated by our results, although the change in chi-square is significant, the lack of change in RMSEA or CFI leads to the conclusion that the model is invariant. Hence, the factor structure is the same for the two language groups, indicating the scale and factors are not sensitive to language (French or English).

**Table 5:** **Fit indices for each hypothesis test of the factor structure of the SAT-PLWHA-S using the RML estimator**

	Hypothesis B	Hypothesis C	Hypothesis D	Hypothesis E
χ2	1826.763*	1841.487*	3719.459*	3889.574*
Δ χ2	-	14.724	1877.972*	170.115*
df	606	626	653	681
CFI	1.000	1.000	1.000	1.000
NNFI	1.032	1.032	1.032	1.032
GFI	0.934	0.928	0.638	0.640
PNFI	0.863	0.892	0.930	0.970
RMSEA	0.000	0.000	0.000	0.000
SRMR	0.043	0.0519	0.149	0.208

*Note.* n = 689. df = degrees of freedom; CFI = comparative fit index; NNFI = non-normed fit index; GFI = goodness-of-fit index; PNFI = parsimony normed fit index; RMSEA = root-mean-square error of approximation; SRMR = standardized root-mean-square residual. *p < 0.05.

### Reliability: internal consistency

The reliability of all factors was high to moderate: concerns about occasional encounters (0.74), avoidance of personal contact (0.79), responsibility and blame (0.77), liberalism (0.69), non-discrimination (0.77), criminalization of transmission (0.69), and confidentiality of serological status (0.59). The overall scale (27 items) was reliable, as indicated by the Cronbach’s alpha of 0.88 for the total scores. Internal consistency was similar for men (0.88) and women (0.88) and for English (0.90) and French speakers (0.88).

### Discriminant validity

The comparisons of stigmatizing attitudes towards PLWHA (mean socre) for different subgroups are presented in Table 6. Overall, the results show satisfactory discriminant validity. The factor and total scores distinguish stigmatizing attitudes between the groups. The individuals with more stigmatizing attitudes towards PLWHA were men, older, born outside Canada, less educated, and more homophobic. They also did not know any PLWHA and had less HIV/AIDS transmission knowledge. However, it is important to note the large effect (Cohen’s d) size for “HIV knowledge” and “homophobia”.

**Table 6:** **Stigmatizing attitudes towards PLWHA: factor and total scores for total sample and different groups**

	(n)	Total score	Cohen’s d	F1	Cohen’s d	F2	Cohen’s d	F3	Cohen’s d	F4	Cohen’s d	F5	Cohen’s d	F6	Cohen’s d	F7	Cohen’s d
Total	1387	2.998	-	3.489	-	3.461	-	3.288	-	2.750	-	2.938	-	2.439	-	2.459	-
Language																	
English	273	2.961	-0.101	3.445	-0.097	3.490	0.056	3.273	-0.030	2.665	-0.144	3.001	0.116	2.477	0.063	2.137*	-0.504
French	1114	3.007		3.500		3.453		3.291		2.771		2.922		2.430		2.537	
Sex																	
Men	698	2.955***	-0.228	3.425***	-0.309	3.361***	-0.222	3.223***	-0.025	2.741	-0.119	2.897*	0.007	2.442	-0.056	2.436	-0.194
Women	689	3.042		3.553		3.561		3.353		2.759		2.978		2.437		2.481	
Age																	
Lower quartile	422	3.063***	0.215	3.540**	0.394	3.560***	0.019	3.270	0.387	2.847***	0.207	3.009**	0.037	2.467	0.387	2.652***	0.335
Upper quartile	311	2.913		3.414		3.299		3.259		2.568		2.868		2.440		2.339	
Country of birth																	
Outside Canada	150	2.916*	-0.230	3.373*	-0.081	3.414	-0.189	3.189*	-0.213	2.611*	-0.024	2.924	-0.020	2.426	-0.228	2.295**	-0.207
Canada	1236	3.009		3.503		3.467		3.300		2.768		2.940		2.441		2.479	
Education																	
Lower quartile	265	2.829***	-0.262	3.387**	-0.307	3.345***	-0.613	3.038***	-0.632	2.469***	-0.456	2.758***	-0.463	2.246***	0.080	2.520	-0.608
Upper quartile	336	3.103		3.534		3.545		3.402		2.933		3.064		2.575		2.453	
Acquainted with PLWHA																	
No	1026	2.956***	-0.299	3.445***	-0.348	3.402***	-0.304	3.241***	-0.222	2.708***	-0.294	2.885***	-0.216	2.397***	-0.001	2.458	-0.362
Yes	360	3.118		3.613		3.627		3.419		2.870		3.083		2.557		2.459	
Knowledge																	
Lower quartile	370	2.744***	-0.909	3.191***	-0.844	3.166***	-0.722	3.055***	-0.693	2.484***	-0.914	2.622***	-0.606	2.208***	-0.237	2.336***	-1.092
Upper quartile	403	3.199		3.683		3.683		3.466		2.965		3.214		2.650		2.529	
Homophobia																	
Lower quartile	303	3.299***	0.892	3.706***	1.176	3.793***	1.319	3.619***	1.191	3.127***	1.086	3.282***	0.773	2.709***	0.500	2.608***	1.645
Upper quartile	406	2.648		3.204		3.084		2.896		2.305		2.589		2.153		2.211	

*Note.* n = 1387. *p < 0.05; **p < 0.01; ***p < 0.001.

Stratified analysis by factor showed a similar trend to the total factor scores, with some minor differences. With the exception of Criminalization of HIV transmission -F7, no significant differences were found between English and French-speaking participants. However, whereas more English speakers were in favour of criminalization (F7), there were no differences for sex, education, or acquaintance with PLWHA. In addition, there was no difference by country of birth on avoidance of personal contact (F2) or non-discrimination (F5). Similarly, there was no age difference in terms of responsibility and blame (F3), and no sex difference on liberalism (F4). In addition, no significant difference was found for sex, age, or country of birth for scores on confidentiality of seropositive status (F6).

### Criterion-related and concomitant validity

Table 7 presents the correlations between stigmatizing attitudes towards PLWHA and male homophobia. Results show that the SAT-PLWHA-S correlates moderately negatively with the homophobia scale (-0.59), with each factor correlating negatively (from -0.50 to -0.35) with the homophobia scale. In contrast, two factors, confidentiality of seropositive status (F6) and criminalization of transmission (F7), show weaker correlation (from -0.24 to -0.29) with homophobia scores.

**Table 7:** **Correlations among stigmatizing attitudes towards PLWHA and male homophobia**

Groups	F1	F2	F3	F4	F5	F6	F7	Total score
All	-.346***	-.404***	-.497***	-.493***	-.391***	-.291***	-.243***	-.585***
French	-.322***	-.377***	-.478***	-.462***	-.361***	-.306***	-.282***	-.570***
English	-.397***	-.503***	-.553***	-.570***	-.497***	-.268***	-.090	-.624***

*Note.* n = 1387. ***p < 0.001.

Table 8 presents the sequential regression analysis of the total mean score on stigmatizing attitudes. Results indicate that the total score on stigmatizing attitudes towards PLWHA is largely explained by homophobia and HIV/AIDS transmission knowledge. These two variables alone account for 42.2% of the variance of the total score on stigmatizing attitudes, whereas the addition of the sociodemographic variables explains only an additional 1.1%. Five independent variables are associated with stigmatizing attitudes towards PLWHA: homophobia, transmission knowledge, acquaintance with PLWHA, years of education, and age. Sex, language spoken at home, and country of birth do not make a significant contribution to explain stigmatizing attitudes.

**Table 8:** **Sequential regression on total scores for stigmatizing attitudes towards PLWHA (n = 1,368)**

BLOCKS	Variables	B	SE B	β	Adjusted R ^2^
BLOCK 1	Homophobia	-.239	.011	-.503	.343***
BLOCK 2	Knowledge	.307	.025	.266	.422***
BLOCK 3	Sociodemographic				
	Sex	-.019	.019	-.021	.433***
	Language: French	-.017	.025	-.015	
	Age	-.002	.001	-.058	
	Country of birth: Canada	-.006	.032	-.004	
	Education	.008	.003	.061	
	Acquaintance with PLWHA	.092	.021	.090	

*Note.* n = 1387. ***p < 0.001.

## Discussion

The aim of this study was to develop and validate the SAT-PLWHA-S through a series of structured steps designed to adjust and improve the scale. The overall factor structure of the final scale is consistent with the sociocognitive conceptualization of stigma developed by the steering committee, it considers not only attitudes (stereotypes, prejudices, discrimination) and values concerning PLWHA, but also social distancing and support for coercive measures [35, 53]. The stronger the values against PLWHA, the greater the social distancing, the support for coercive measures, and the potential for stigma. These conceptual dimensions are clearly and distinctly expressed in the scale, and present a confirmed structure.

Conceptually concerns about occasional encounters (F1) and avoidance of personal contact (F2), these two attitudes come under the umbrella of prejudices, whereby people erroneously fear interacting with PLWHA in diverse situations [75]. In agreement with the most recently published data, the rather high factor scores on these two factors show that Quebec’s population does not appear to hold unfavourable attitudes towards contact with PLWHA [42].

Similar to the present study’s examination of responsibility and blame (F3), many researchers have shown that people tend to blame PLWHA for their condition more than they seek to avoid them [3, 42, 48, 76].

Non-discrimination (F5) appears to be a very important aspect of stigma, with potentially negative consequences for PLWHA [27]. Given that this factor constitutes a distinct component of the scale, it may be concluded that attitudes that favour discrimination should be included in a complete conceptualization of stigmatizing attitudes, as suggested by Pescosolido et al. [77].

The last two factors operationalize the most severe form of discrimination, e.g. support for coercive measures [53]. “Confidentiality of serological status” - F6 includes three items that measure support for the confidentiality of HIV/AIDS, and “Criminalization of HIV transmission” - F7 (3 items) measures criminalization of HIV/AIDS *transmission*. Identifying specific, meaningful concepts and defining corresponding subscales that reflect current situations improves the scale validity over scales that address outdated coercive measures, such as quarantine, refusal of admittance to countries, public identification of PLWHA and mandatory screening tests [61, 78, 79]. Hence, it is more sensitive to new, more subtle forms of stigmatization [50]. The scores obtained on these two factors suggest that the attitudes of Quebec’s population concerning coercion are moderate, concurring with studies in the United States by Herek and colleagues [61]. It may be appropriate to consider the tendency to support coercive measures against PLWHA in the study of HIV/AIDS stigmatization and whether this new form of stigma occurs cross-culturally.

This new scale may be useful in several ways. First, it could be used as a surveillance tool to monitor HIV/AIDS stigmatizing attitudes at the population level, but also to assess the effectiveness of awareness campaigns. Second, the scale could be used in other industrialized countries similar to Quebec (United States, England, Australia, France) since structural invariance between the English and French versions has been demonstrated. The use of the scale in other contexts could help to accurately compare different populations. Third, used in combination with other behavioral measures (e.g. HIV/AIDS testing), the scale could lead to a better understanding of the impact of stigma on health behaviors. The development of this scale also has potential implications for future research. Dimensions covered by the SAT-PLWHA-S reflect the new reality of HIV and its subtle forms of stigmatization. Future research may explore the influence of those new dimensions and determine if they are more harmful for PLWHA.

Although the results demonstrate that the SAT-PLWHA-S is a valid instrument for measuring stigmatizing attitudes towards PLWHA, this study contains certain limitations. First, as is often the case with telephone surveys, the sample may not be representative of the population because it excluded people who do not have a residential landline phone [80]. Unfortunately, the non-respondent profile was not documented, it is thus hard to estimate the extent to which non-response could have biased the results. Second, it is also possible that the results were biased by a degree of social desirability due to the socially sensitive topic. Some respondents may have modified their responses towards less stigma than they actually felt [81]. Furthermore, this study considers stigma from a sociocognitive perspective, which does not allow a complete accounting of the complex issues involved. To thoroughly examine this subject in all its complexity, the SAT-PLWHA-S should be used in combination with other structural data and measures, such as discourse analyses of laws, public policy, and the media. Although this study established some important psychometric properties of the SAT-PLWHA-S, stigma changes over time, as the perception of HIV/AIDS itself evolves. Therefore, this scale would require periodic review to update the content and revalidate revised versions.

Although the need has been stressed to relate measures of HIV/AIDS stigma to measures of associated concepts, few studies have assessed criterion-related validity in this manner [53]. An innovative aspect of the present study is the assessment of concomitant validity through associations between stigmatizing attitudes, homophobia, and HIV/AIDS transmission knowledge. The association between stigmatizing attitudes and homophobia could also indicate that PLWHA are subject to numerous forms of stigmatization [32, 82]. In addition, the discriminant validity analysis shows that the SAT-PLWHA-S can also discriminate between the responses of different subgroups. Thus, individuals who have more stigmatizing attitudes towards PLWHA are more homophobic, have less HIV/AIDS transmission knowledge, are not acquainted with PLWHA, have less education, and are older. Furthermore, the results show no differences between French and English speakers.

## Conclusion

Developed in collaboration with a steering committee involving a number of key stakeholders in the field, the SAT-PLWHA-S reflects current concerns surrounding HIV/AIDS. It enables bridging the gap between emerging practical issues and conceptual considerations. In this sense, it contributes to a deeper understanding of the complex concept of stigma. The SAT-PLWHA-S should enable a better appreciation of how PLWHA are stigmatized. In addition, the data collected with this scale can be used to tailor interventions aimed at more effectively adressing stigma.

## Electronic supplementary material

Additional file 1: **Exploratory factor analysis of the SAT-PLWHA-S.** (DOC 96 KB)
