# Supplementary material for: Stigmatizing attitudes towards people living with HIV/AIDS: validation of a measurement scale
Source: BMC Public Health. 2014 Dec 4;14:1246. doi: 10.1186/1471-2458-14-1246 (PMC4289343; doi:10.1186/1471-2458-14-1246)
Supplement: Supplementary file 1 — Additional file 1: Exploratory factor analysis of the SAT-PLWHA-S. (DOC 96 KB) [file 12889_2014_7404_MOESM1_ESM.doc]

## Table 2 - Exploratory factor analysis of the SAT-PLWHA-S: Final solution, completely standardized for 7 oblique factors

| Item No. |  | Factor loadings | | | | | | | Communalities |
| --- | --- | --- | --- | --- | --- | --- | --- | --- | --- |
|  | Item description | F1 | F2 | F3 | F4 | F5 | F6 | F7 |  |
|  | **F1: Concerns about occasional encounters** |  |  |  |  |  |  |  |  |
| 1 | Being around someone who has AIDS does not bother me. | .635 |  |  |  |  |  |  | .594 |
| 2 | I would not be worried for my health if a co-worker had AIDS. | .690 |  |  |  |  |  |  | .601 |
| 3 | It would not bother me if there was a boarding house for people with AIDS on my street. | .315 |  |  |  |  |  |  | .349 |
|  | **F2: Avoidance of personal contact** |  |  |  |  |  |  |  |  |
| 4 | I could not be friends with someone who has AIDS. |  | -.717 |  |  |  |  |  | .496 |
| 5 | I would limit my contact with a person whom I know is infected with AIDS. |  | -.608 |  |  |  |  |  | .568 |
| 6 | I would not hug someone with AIDS. |  | -.791 |  |  |  |  |  | .681 |
|  | **F3: Responsibility and blame** |  |  |  |  |  |  |  |  |
| 7 | People who use injectable drugs deserve to have AIDS. |  |  | -.696 |  |  |  |  | .450 |
| 8 | My support for a person living with AIDS depends on how the person was infected. |  |  | -.433 |  |  |  |  | .334 |
| 9 | I am disgusted by persons who were infected during homosexual relations. |  |  | -.326 |  |  |  |  | .475 |
| 17 | People who are infected with the AIDS virus because they have not used a condom deserve what they get. |  |  | -.644 |  |  |  |  | .465 |
| 29 | People with AIDS have only themselves to blame. |  |  | -.577 |  |  |  |  | .337 |
| 39 | Most people with AIDS are responsible for having their illness. |  |  | -.447 |  |  |  |  | .297 |
|  | **F4: Liberalism** |  |  |  |  |  |  |  |  |
| 10 | To fight AIDS, it is necessary that young people not have sex. |  |  |  | .393 |  |  |  | .310 |
| 11 | Reinforcement of traditional sexual values will help to control AIDS. |  |  |  | .516 |  |  |  | .334 |
| 12 | The arrival of AIDS is linked to the fact that people have more sexual freedom. |  |  |  | .559 |  |  |  | .375 |
| 13 | The spread of AIDS is linked to the decline of moral values. |  |  |  | .650 |  |  |  | .571 |
|  | **F5: Non-discrimination** |  |  |  |  |  |  |  |  |
| 14 | People who have AIDS should have the right to work serving the public, as waiters-waitresses, cooks, hairdressers, etc. |  |  |  |  | .703 |  |  | .531 |
| 15 | Children who are infected with the aids virus should be able to go to day-care. |  |  |  |  | .647 |  |  | .510 |
| 16 | Doctors with AIDS should be allowed to go on working with their patients. |  |  |  |  | .655 |  |  | .428 |
| 19 | People infected with the aids virus should be allowed to immigrate to Canada. |  |  |  |  | .485 |  |  | .359 |
| 23 | If I had a roommate and discovered he was infected with the AIDS virus, it would not bother me. |  |  |  |  | .478 |  |  | .418 |
|  | **F6: Confidentiality of serological status** |  |  |  |  |  |  |  |  |
| 27 | I have the right to know if someone around me is infected with the AIDS virus. |  |  |  |  |  | .475 |  | .387 |
| 32 | When a screening test indicates that someone is infected with the AIDS virus, the result should remain confidential. |  |  |  |  |  | .573 |  | .356 |
| 41 | Doctors should report the names of people with AIDS to the government. |  |  |  |  |  | .473 |  | .394 |
|  | **F7: Criminalization of transmission** |  |  |  |  |  |  |  |  |
| 26 | Transmitting the AIDS virus should be punishable by law. |  |  |  |  |  |  | .551 | .335 |
| 31 | People who know they are infected with the AIDS virus and who transmit the virus are criminals. |  |  |  |  |  |  | .598 | .385 |
| 34 | Transmitting the AIDS virus is a crime. |  |  |  |  |  |  | .818 | .698 |

*Note.* n=681.
